# The active mechanical characteristics of arterial smooth muscle during aneurysm remodeling

**DOI:** 10.3389/fbioe.2025.1560193

**Published:** 2025-03-26

**Authors:** Wenqi Qin, Dan Qiao, Mingming Ren, Xiaoqiang Ye, Guanghao Yu, Guangxin Chen, Jian Xing, Wei Ma, Miao Yu, Xiaohuan Yuan, Kunfu Ouyang, Wenchang Tan, Dongliang Zhao

**Affiliations:** ^1^ School of Life Sciences, Mudanjiang Medical University, Mudanjiang, Heilongjiang, China; ^2^ Department of Pathology, Sir Run Run Shaw Hospital, Zhejiang University, Hangzhou, Zhejiang, China; ^3^ Department of Cardiovascular Surgery, Peking University Shenzhen Hospital, Shenzhen, Guangdong, China; ^4^ Medical Image College, Mudanjiang Medical University, Mudanjiang, Heilongjiang, China; ^5^ Basic Medical School, Mudanjiang Medical University, Mudanjiang, Heilongjiang, China; ^6^ Peking University Shenzhen Graduate School, Shenzhen Bay Laboratory, Shenzhen, Guangdong, China; ^7^ PKU-HKUST Shenzhen-Hong Kong Institution, Shenzhen, Guangdong, China; ^8^ Department of Mechanics and Engineering Science, College of Engineering, Peking University, Beijing, China

**Keywords:** abdominal aortic aneurysm, active mechanical characteristics, growth remodeling, smooth muscle, vasospasm

## Abstract

**Introduction:**

Abdominal Aortic Aneurysm (AAA) is a common vascular disease characterized by progressive expansion and remodeling of the aortic wall. However, with the gradual expansion of blood vessels, the walls of blood vessels cannot withstand the tension and rupture, jeopardizing people’s health.

**Methods:**

The aim of the experiment was to establish an abdominal aortic aneurysm model in rats by applying porcine pancreatic elastase externally, to measure the diameter and thickness of blood vessels as well as hemodynamics using animal ultrasound, to measure the active contraction of blood vessels, the rate of contraction, and the contraction stress using vascular mechanics equipment, and to observe the pathological changes in the process of AAA growth using vascular pathological staining.

**Results:**

This study revealed that with the escalation of the inflammatory response, there is a breakdown of elastic fibers and collagen fibers, leading to a decrease in the active contraction force of the arteries. However, it was observed that by alleviating the inflammation, there was a notable enhancement in the active contraction force of the arteries.

**Discussion:**

To describe the development process of AAA from a biomechanical point of view, to reveal the histopathological mechanism, and thus to identify the theoretical basis for clinical treatment.

## 1 Introduction

Cardiovascular and cerebrovascular diseases stand as the leading threats to life and health. High-incidence conditions such as vasospasm, aneurysms, arterial dissections, and atherosclerosis are intricately linked to the functionality of vascular smooth muscle (Cao et al., 2022; Krishnan et al., 2024). While the endothelium does not directly influence the pressure conditions within blood vessels, vascular smooth muscle cells (VSMCs) act as the effectors and integrators of various inputs, including pressure, nerve signals, endothelial cell interactions, circulating vasoactive substances, and autologous hormones. The active contraction of vascular smooth muscle cells plays a pivotal role in regulating the pressure within the circulatory network (Lu et al., 2021). As individuals age, molecular and mechanical alterations lead to pathological remodeling of arterial smooth muscle. However, the active contraction mechanical characteristics of vascular smooth muscle are often overlooked in the remodeling process.

Pressure myography and wire myography represent two essential methods for assessing the mechanical behavior of blood vessels. Pressure myography primarily examines the passive mechanical properties of blood vessels, delving into changes in arterial tissue stiffness during growth and remodeling (Ma et al., 2008). This technique permits fluid-solid coupling analysis and the discovery of biophysical laws. On the other hand, wire myography serves as a valuable and reliable tool for evaluating myogenic active responses (del Campo and Ferrer, 2015). It can assess endothelial function, vasoconstriction or vasodilation properties of blood vessels, as well as the vasomotor responses to various stimuli or substances, shedding light on the involvement of different factors and molecular pathways in controlling vascular tone. Wire myography has been instrumental in studying the stability of atherosclerotic plaques (Chen et al., 2023), NETosis-induced hypertension and vascular dysfunction (Krishnan et al., 2024), and the gender-specific effects of a high-fat diet on aortic inflammation and dysfunction (Tran et al., 2023). While the passive behavior of arterial vessels predominantly focuses on the macroscopic mechanical response of smooth muscle cells, collagen fibers, and their spatial arrangement, detailed investigations into changes in active dynamics during aortic pathological remodeling are crucial for elucidating the role of smooth muscle cells, yet such studies are lacking.

Abdominal aortic aneurysm (AAA) represents a prevalent vascular disorder characterized by progressive dilatation and remodeling of the aortic wall. Typically defined as a permanent dilation of the abdominal aorta, with a diameter at least 50% larger than the normal expected diameter, AAA poses a significant challenge in early diagnosis and treatment due to its initially asymptomatic nature (Zhang et al., 2024). As the vascular wall gradually dilates, the arterial wall weakens, eventually rupturing under pressure from the vascular lumen, significantly increasing patient mortality rates and posing a substantial threat to human life, causing 150,000 to 200,000 deaths worldwide annually (Davis et al., 2015; Gao et al., 2023). Risk factors for AAA primarily include advanced age, smoking, hypertension, hyperlipidemia, and genetic predisposition (Pinard et al., 2019; Cho et al., 2023; Gao et al., 2023). Presently, there is a lack of approved effective medications to prevent the growth or rupture of abdominal aortic aneurysms.

The rupture of an AAA is associated with the dilation of the arterial diameter, resulting from progressive dilatation and weakening of the aortic endothelium, mesothelium, and integument. Pathophysiological mechanisms driving aneurysm formation primarily involve aortic wall inflammation, elastin degradation, oxidative stress, smooth muscle cell phenotypic changes and dysfunction, extracellular matrix degradation, and neovascularization (Lu et al., 2021; Qian et al., 2022). Alterations in vascular components further impact the function and behavior of blood vessels. Changes in structural proteins play a vital role in the mechanical behavior of the vessel wall during aneurysm growth and remodeling (Cheng and Wagenseil, 2012). Arterial tissues exist within a complex hemodynamic environment, and once the mechanical stress on the vascular wall surpasses its strength, an aneurysm may rupture (Wagenseil and Mecham, 2009). Under the hemodynamic pressures of hypertension, the vascular wall tissue of an AAA undergoes remodeling, leading to wall thinning, compensatory remodeling of elastic and collagen fibers, increased mechanical properties, but reduced strength (Cheng and Wagenseil, 2012). Various studies have explored the mechanical properties of AAA tissues in patients, analyzing fiber remodeling during aneurysm rupture and the effects of stent placement (Annambhotla et al., 2008; Kandail et al., 2014; Sommer et al., 2016; Sugita and Matsumoto, 2018). The active mechanical characteristics and vascular strength work together to uphold vascular function. Inadequate vascular strength could potentially constrain the contractile capacity of smooth muscle and impact the regulatory function of blood vessels (Lu et al., 2017; Zhao et al., 2020). While the mechanical properties of aortic vessels have been extensively studied, the changes in mechanical and dynamic properties during vascular remodeling under pathological conditions have received less attention.

**FIGURE 1:**
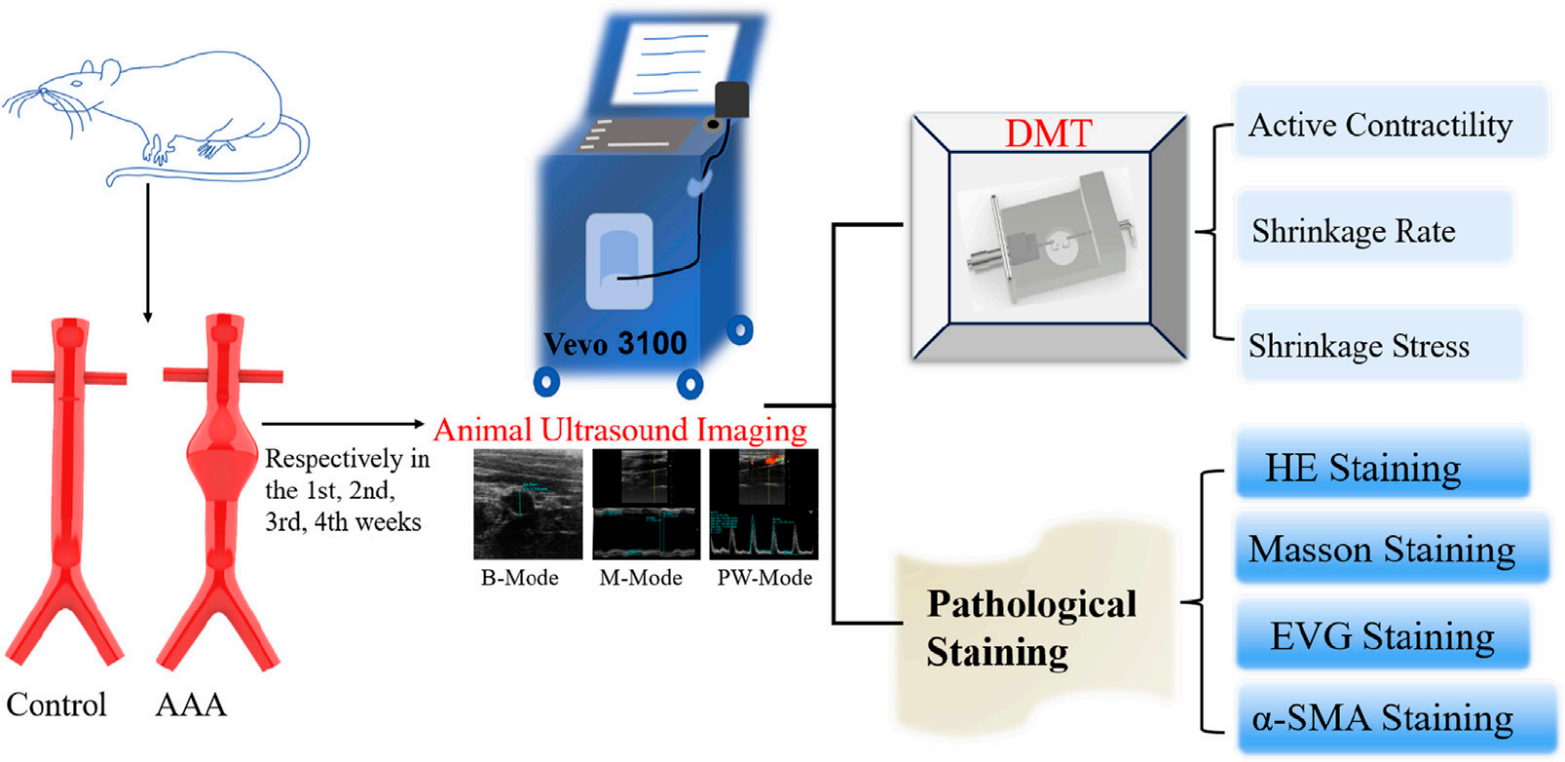
Overall idea of the subject.

In this study, porcine pancreatic protein was utilized to induce the formation of an AAA model in Sprague-Dawley (SD) rats, allowing for the exploration of the primary dynamic characteristics of vascular remodeling over a period of 1–4 weeks. Ultrasound imaging was employed to macroscopically observe the morphology and hemodynamic alterations of AAA, while histological techniques such as Hematoxylin and Eosin (HE), Masson’s trichrome, Elastica van Gieson (EVG), and alpha-smooth muscle actin (α-SMA) staining were conducted on the AAA tissue to microscopically examine structural changes. Wire myography was employed to assess the primary dynamic responses during arterial lesions at 1, 2, 3, and 4 weeks, elucidating the impact of various vascular components on the primary dynamic behavior of blood vessels. The active mechanical properties, including vasoconstriction capacity, contraction stress, and vasoconstriction rate, were measured using wire myography. The research framework of this study is illustrated in Figure 1.

## 2 Materials and methods

### 2.1 Experimental protocol

20 male 6-week-old Sprague-Dawley (SD) rats were purchased from Viton Lever Ltd. and all rats weighed about (250 ± 20) g. The rearing conditions were SPF grade animal house of Shenzhen Bay Laboratory, and the ambient temperature of the SPF animal house was 22°C ± 1°C and the humidity was 60% ± 5%, and all the experimental animals were acclimatized to be fed for 1 week for experiments, and all the experimental operations were approved by the Shenzhen Bay Laboratory Animal Protection and Ethics Committee (Certificate No. AETWC202101).

### 2.2 Abdominal aortic aneurysm model

Male Sprague-Dawley (SD) rats were completely randomized into Control and AAA postoperative 1-week, 2-week, 3-week, and 4-week groups using a randomized numerical control table, recorded as AAA-1w, AAA-2w, AAA-3w, and AAA-4w groups, respectively, with four animals in each group, and the animals were fasted 24 h before the experiment. We performed AAA modeling in rats by external application of porcine pancreatic elastase to the abdominal aorta as previously described (Romary et al., 2019; Tyerman et al., 2019; Xue et al., 2022). All animals were anesthetized with 2.5% aflutidine *via* intraperitoneal injection at a dose of 10 mL/kg. The animal was immobilized in the supine position, the abdominal aorta was exposed, and the main segment of the unbranched abdominal aorta was freed for 1.5 cm, and a gelatin sponge was compressed and shaped to pass through the gap between the abdominal aorta and the inferior vena cava. The area was infiltrated with drops of porcine pancreatic elastase (SIGMA-E1250) for 30 min and wrapped in prepared sterile rubber gloves to protect the inferior vena cava and surrounding tissues. Saline rinses were repeated 3 times to prevent protease damage to the surrounding tissues to form adhesions. Postoperative animals were kept in separate cages in a warm environment. Normal diet was given after defecation. Control group: sham operation group, PBS was applied under the same experimental conditions.

### 2.3 Animal ultrasound imaging

As previously described, we performed animal ultrasound imaging of animals at various cycles after surgery (Phillips et al., 2015; Melin et al., 2022), the Control and AAA groups were treated at 1, 2, 3, and 4 weeks postoperatively, respectively, by fasting for 24 h in advance and pre-inducing anesthesia in the animals with an animal respiratory gas anesthesia machine. Subsequently, the anesthesia was changed to mask anesthesia to ensure that the animal’s heart rate was 300–500 beats/min, which was the optimal anesthesia state and depth of anesthesia. The abdomen was cleaned of hair, ultrasound couplant was squeezed and spread over the abdomen, and the abdominal aortic diameter, wall thickness, and hemodynamics were examined with a Vevo 3,100 imaging system (VisualSonics) in B-mode, M-mode, and pulsed Doppler mode. Ensure that there is no air and gap between the ultrasound probe and the coupler, and ensure that the probe position of each mouse is the same as the angle of the final imaged blood vessel, and that the blood vessel is imaged as a horizontal plane as the imaging standard, in order to reduce the imaging error and standardize the standard. Each one was imaged according to the above procedure and by the same operator from start to finish to minimize errors in handling by different operators. All images taken were retained and data recorded for later data analysis.

The captured ultrasound images were analyzed using Vevo3100 software, and the maximum diameter of the vessels in the short-axis and long-axis directions as well as the dilatation of the vessels in the B-mode were calculated using the straight-line distance measurement tool, and the changes in the wall thickness in the M-mode were also calculated, and the mean flow velocity, peak flow velocity, resistance index, and beat-to-beat index were computed on the basis of the velocity waveforms on the pulsed Doppler images.

Artrial diameter refers to the width of the vessel lumen. Abnormal changes in vascular diameter can indicate dilation, stenosis, or occlusion, reflecting the vascular health status.

Artrial wall thickness is an indicator of vascular health. In cases of vasculitis, an increase in giant cells can lead to significant thickening of the vessel wall, which can be detected by ultrasound.

Blood flow velocity is a key parameter reflecting the hemodynamics within a vessel. Changes in blood flow velocity can indicate the patency of the vessel and the hemodynamic status, directly affecting tissue perfusion. Measurements and analyses are conducted using spectral Doppler ultrasound.

Resistance Index is a hemodynamic parameter calculated from flow velocity using spectral Doppler ultrasound. It represents the ratio of the change in blood flow velocity over the cardiac cycle to the maximum end-systolic flow velocity. RI is used to assess the resistance in distal vessels. It is calculated as RI = (PSV - EDV)/PSV, where PSV is the peak systolic velocity and EDV is the end-diastolic velocity, which is the lowest velocity at the end of diastole. RI primarily reflects vascular resistance, indicating the resistance encountered by blood flow. A higher RI indicates greater resistance. The end-diastolic phase is when the heart’s contractile force is weakest in propelling blood flow. When vascular resistance increases, both systolic and diastolic blood flow are suppressed, with the end-diastolic phase being more affected. If resistance further increases, diastolic flow may disappear, leaving only a systolic peak, and when EDV becomes zero, the resistance index is 1. With further resistance increase, PSV will also decrease.

### 2.4 Isolated vascular tone measurement

**FIGURE 2:**
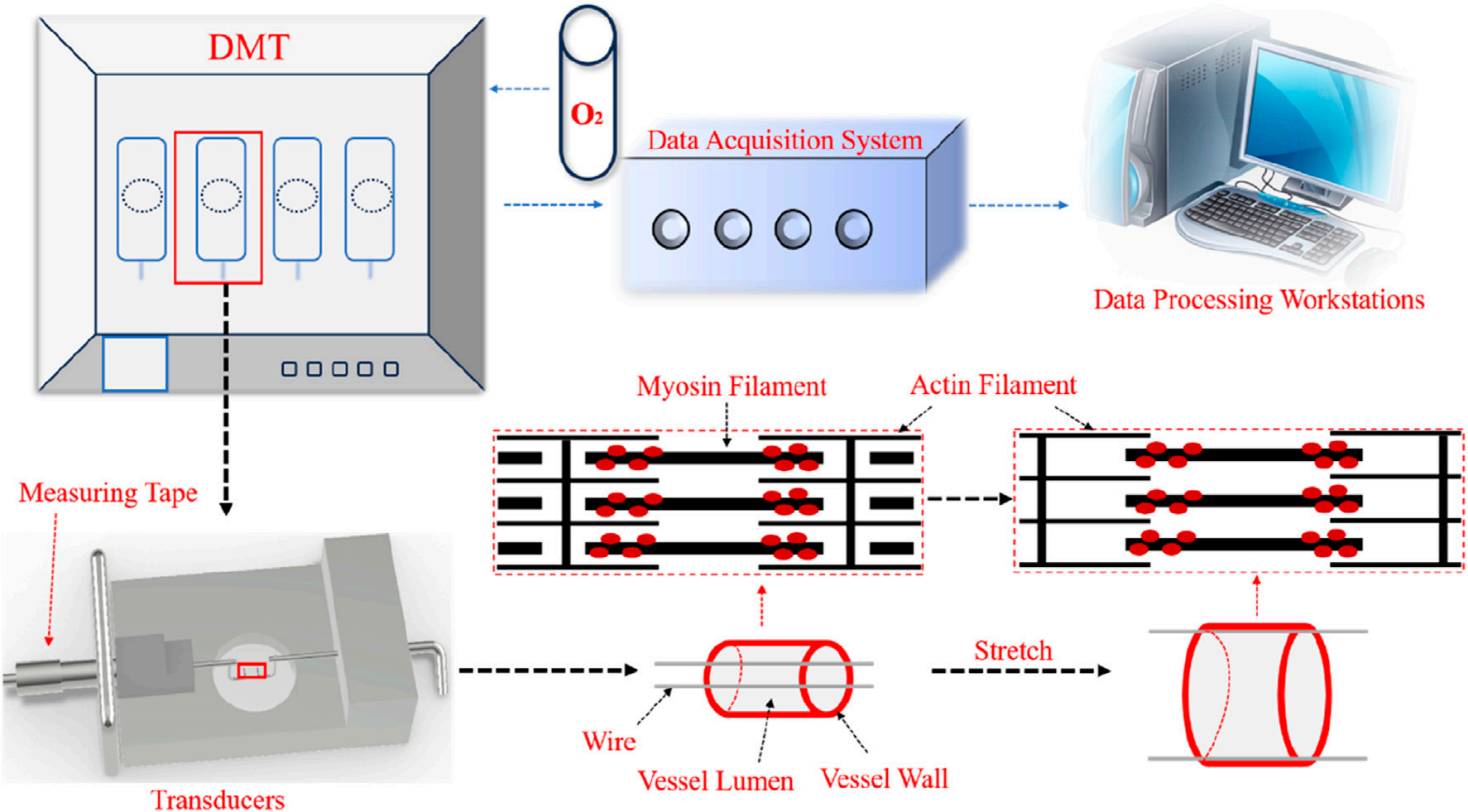
Schematic diagram of DMT vascular electromyography.

Wire myography was performed as previously described (del Campo and Ferrer, 2015; Morris et al., 2001; Ma et al., 2008). Briefly, a surgical segment of the abdominal aorta was obtained from a rat and cleared of fat and connective tissue, and the aorta was sliced into two 2-mm-long rings mounted on two steel needles in a wire myograph system (620M, DMT). And immersed in 37°C PSS buffer (130 mM NaCl, 4.7 mM KCl, 1.18 mM KH_2_PO_4_, 1.17 mM MgSO_4_ 7H2O, 14.9 mM NaHCO_3_, 5.5 mM Glucose, 0.0026 mM EDTA, 1.16 mM CaCl_2_) with constant outgassing (95% O_2_ and 5% CO_2_). The vascular ring was mounted on a steel needle electromyographically, and the diameter-tension relationship was determined by gradually stretching the tissue to increase its passive diameter by increasing the distance between the wires. The force and distance between the steel needles were recorded at each step. The diameter of the vessel segment was calculated using Laplace’s equation when the force equaled 100 mmHg. The arterial segments were set at their optimal tension and held for the remainder of the experiment. After stabilizing equilibrium tension for 1 h, the arteries were exposed to 60 mM KCl to check their functional integrity. The endothelial inhibitors 10 μM indomethacin and 100 μM N-nitro-L-arginine were subsequently added to inhibit endothelial cell activity and function. Changes in vasoconstrictor capacity were studied by exposing segments to increasing doses of norepinephrine (from 1 nM to 10 μM), and the data are expressed as a percentage of contractions induced by 60 mM KCl. An overview of the DMT instrument and the principles of operation associated with the experiment are shown in Figure 2.

### 2.5 Calculation of vascular contraction rate

**FIGURE 3:**
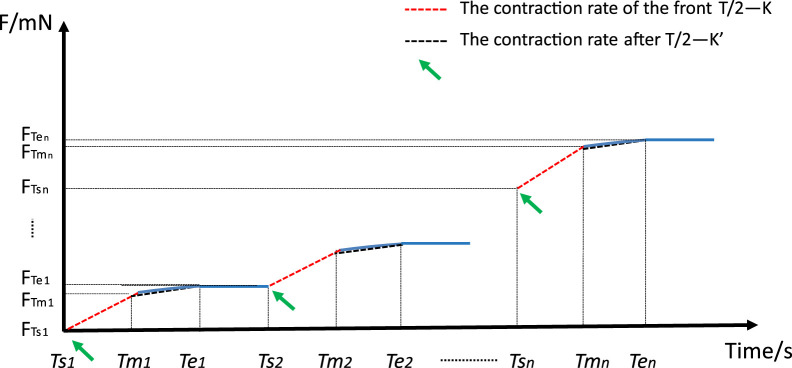
Graphical representation of shrinkage rate analysis of mechanical data.

In order to obtain the contraction of the blood vessel after the addition of the contractile drug, Figure 3 shows a graphical illustration of the contraction rate analysis of the mechanistic data, in which the time point of the addition of the drug is denoted as *Ts*, the time point of reaching the plateau phase is denoted as *Te*, and the intermediate time point between *Ts* and Te is denoted as *Tm*, i.e., the segment from *Ts* to *Tm* is denoted as the first half of the contraction curve, and the segment from *Tm* to Te is denoted as the second half of the contraction curve. Each section of the curve is scaled to take 10 points at the same time intervals, the corresponding mechanical readings are recorded, and a straight line is fitted to these data points to derive the slope of the straight line, which is denoted as K for the first half of the curve, and K′ for the second half of the curve, and the following steps are the procedure for the calculations:

$$K_{1}=\frac{F_{T_{m1}}-F_{T_{s1}}}{T_{m1}-T_{s1}}$$

$$K_{1}^{\prime }=\frac{F_{T_{e1}}-F_{T_{m1}}}{T_{e1}-T_{m1}}$$

$$K_{n}=\frac{F_{T_{\text{mn}}}-F_{T_{\text{sn}}}}{T_{mn}-T_{sn}}$$

$${\;K^{\prime }}_{n}=\frac{F_{T_{\text{en}}}-F_{T_{\text{mn}}}}{T_{en}-T_{mn}}$$

### 2.6 Calculation of vasoconstrictive stress

Stresses are generated when an object is deformed due to an external cause, creating internal forces interacting between parts within the object to resist this external cause and attempting to restore the object from its deformed position to its pre-deformed position (Rodríguez et al., 2008; Malkawi et al., 2010; Niestrawska et al., 2019). Stress (σ) is the force exerted on an object divided by the surface area of the object on which the force is applied. In this experiment, the length of the blood vessel sample L was 2 mm, the thickness of the blood vessel wall was measured by ultrasound to be D, and the contraction force obtained through the vascular tension curve to be F. Therefore, the stress was calculated as the following equation:

$$\sigma =\frac{F}{L*D}$$

### 2.7 Histology and immunofluorescence

The abdominal aortas of experimental rats were fixed with 4% paraformaldehyde and then rinsed with PBS, and the tissues were subsequently dehydrated and paraffin-embedded, and the abdominal aortas were cut into 4-μm serial sections, which were affixed to slides to facilitate the subsequent pathological staining with HE, Masson, EVG, and α-SMA. As mentioned earlier we will evaluate the abdominal aortic tissue structure (Cullen et al., 2019; Sharma et al., 2019; Chen et al., 2020; Li et al., 2020; Melin et al., 2022).

HE Staining Kit (Beyotime Biotechnology) is used to observe the inflammation of the abdominal aorta and the morphological changes of the abdominal aorta. Masson staining to assess vascular collagen proliferation was performed using the Masson trichrome staining kit (Solarbio), in which reichhorn red was used to stain muscle fibers and aniline blue was used to stain collagen fibers in order to observe the collagen content of the tissue. EVG staining was performed to observe the breakage of vascular elastic fibers, and collagen fiber-elastic fiber composite staining kit (Solarbio) was used to stain elastic fibers in the vessels. α-Smooth muscle actin (α-SMA) was used to assess the content of vascular smooth muscle cells, and we performed immunofluorescence staining of vascular sections by first incubating the primary antibody (ab7817, Abcam) diluted at 1:1,000 overnight at 4°C, then incubating the secondary antibody (ab1150113, Abcam) at 1:200 dilution and staining the nuclei with DAPI. All slides were viewed under an Olympus pathology scanner and analyzed for percentage of positive markers using ImageJ software.

### 2.8 Statistical analysis

Data were analyzed using GraphPad Prism version 8.0.2 software (GraphPad Software Inc., San Diego, CA, United States), and between-group comparisons were made using a one-way analysis of variance (ANOVA), in which vasovagal active constriction analyses were analyzed using a paired t-test. Statistical significance was defined as a critical value of p-value <0.05, and all experimental data were expressed as mean ± standard deviation of the mean ( $\overset{®}{X}\pm SD$ ).

## 3 Results

### 3.1 Abdominal aortic aneurysm modeling

**FIGURE 4:**
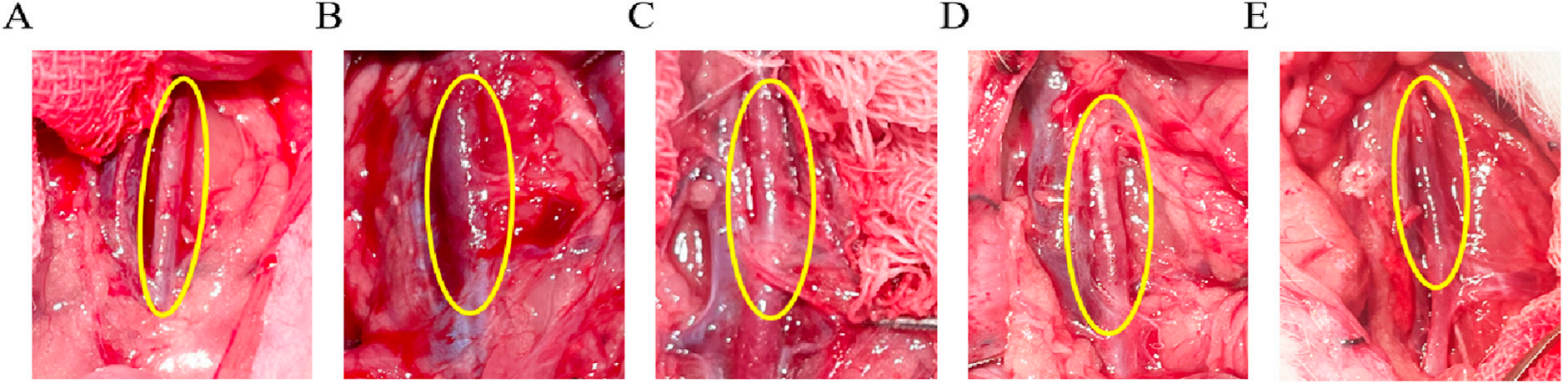
Morphological observation of rat abdominal aorta, the area circled in yellow is the surgical area. **(A)** Control group images. **(B**–**E)** are AAA-1w, AAA-2w, AAA-3w, and AAA-4w images, respectively.

Arterial diameters were measured with vernier calipers at 1.67 ± 0.14 mm in the Control group (Figure 4A), and measuring AAA-1w arterial diameter (Figure 4B) 1.85 ± 0.24 mm, AAA-2w arterial diameter (Figure 4C) 2.04 ± 0.29 mm, AAA-3w arterial diameter (Figure 4D) 2.61 ± 0.06 mm, and AAA-4w arterial diameter (Figure 4E) 1.91 ± 0.04 mm. The dilatation rate was expressed as (post-surgical artery diameter - pre-surgical artery diameter)/(pre-surgical artery diameter) × 100. The artery dilatation rate in the AAA group was 52% in the second week, and the artery dilatation rate was 74% in the third week, with a dilatation rate of 50% or more suggests that the AAA modeling was successful. Morphological results showed that the tissue surrounding the abdominal aorta in Control group was well defined without adhesions. The diameter of the abdominal aorta in AAA group expanded slowly from week one to week 3, and expanded to the maximum in week 3, and then the arterial condition recovered slightly in week 4. In the AAA group, the abdominal aorta was adherent to the surrounding tissues, with poorly defined boundaries, and a gradual transition of the arteries from a dilated state to a normal tissue morphology was observed (Figure 4).

### 3.2 Animal ultrasound imaging analysis

#### 3.2.1 Analysis of ultrasound results within the control group

Firstly, ultrasound measurements were performed 1, 2, 3, and 4 weeks after the Control group’s sham operation to analyze the diastolic and systolic internal diameters, the thickness of the vessel wall, the mean and peak blood flow velocities, as well as the resistance index and pulsatility index of the group, as shown in Table 1. After statistical analysis, there was no statistical difference between the groups in each ultrasound parameter, and the time period after the Control group’s sham operation would not have any impact on the abdominal aorta. The effect of age on the abdominal aorta in the Control group was excluded, so the experimental data of the Control group 4 weeks after sham operation selected by vascular mechanics and pathological staining in the back were compared with those of the AAA group.

**TABLE 1:** Comparison of diastolic diameter (Dd), systolic diameter (Ds), wall thickness, mean velocity (Mean vel), peak velocity (Peak vel), resistance index (RI) and pulsatility index (PI) within the Control group (Mean ± standard deviation).

	Dd (mm)	Ds (mm)	Wall thickness (mm)	Mean vel (mm/s)	Peak vel (mm/s)	RI	PI
Control-1w	1.98 ±0.24	1.46 ±0.12	0.20 ±0.01	175.42 ±20.26	305.74 ±64.2	0.87 ±0.11	1.59 ±0.34
Control-1w	1.8 ±0.22	1.69 ±0.08	0.21 ±0.01	164.27 ±6.93	304.81 ±15.47	0.94 ±0.07	1.74 ±0.11
Control-1w	1.98 ±0.28	1.76 ±0.07	0.23 ±0.02	156.19 ±31.65	305.59 ±42.46	0.9 6 ± 0.06	1.89 ±0.24
Control-1w	1.86 ±0.19	1.67 ±0.14	0.22 ±0.04	192.2 ±42.65	340.28 ±77.01	0.92 ±0.07	1.59 ±0.16
P-value	ns	ns	ns	ns	ns	ns	ns

#### 3.2.2 Abdominal aortic diameter measurement

**FIGURE 5:**
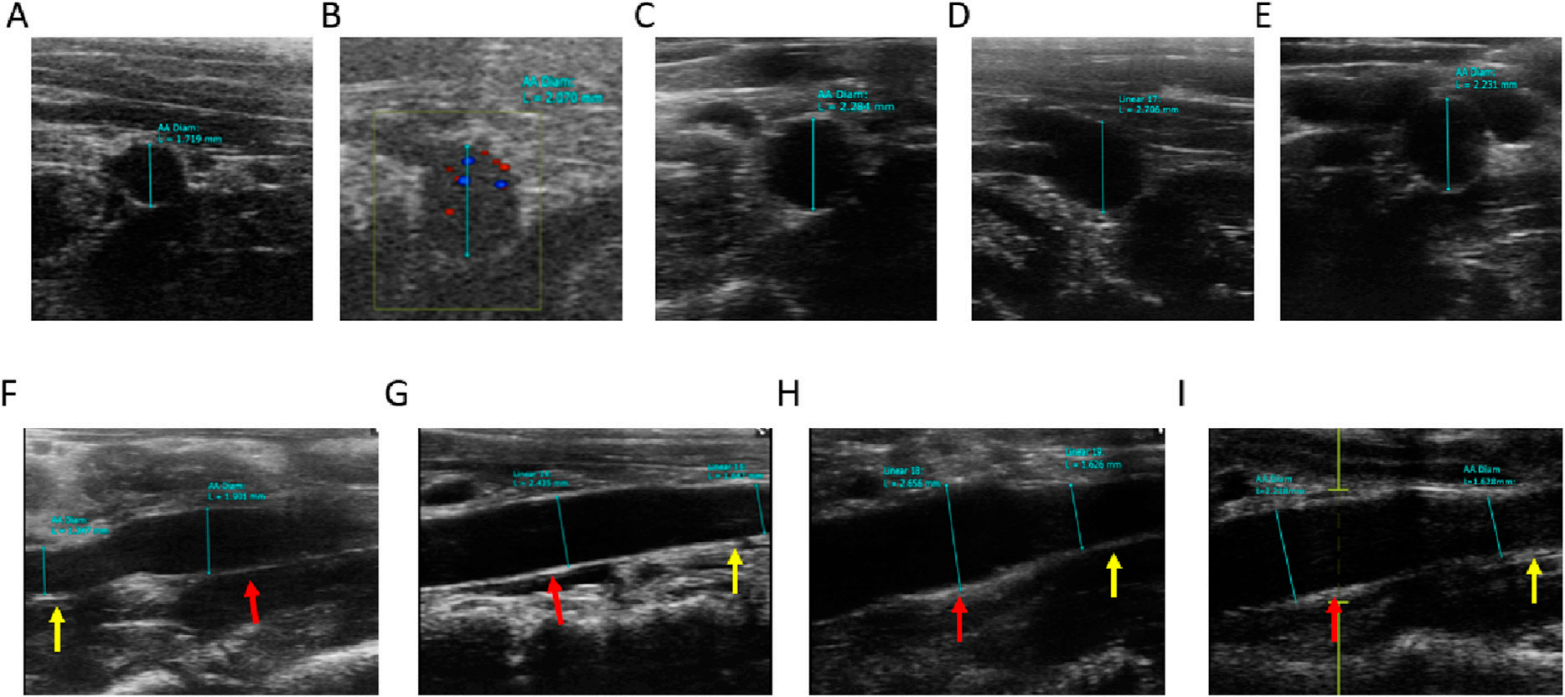
Ultrasound measurement of abdominal aortic vessel diameter, red arrows indicate dilated areas, yellow arrows indicate normal areas. **(A)** Control group short-axis ultrasound images. **(B**–**E)** are short-axis ultrasound images of AAA-1w, AAA-2w, AAA-3w, and AAA-4w, respectively. **(F**–**I)** are long-axis ultrasound images of AAA-1w, AAA-2w, AAA-3w, and AAA-4w, respectively.

Vessel wall diameters in the short and long axes of the vessels at end-diastole were measured in B-mode imaging mode. The mean diameter of the local abdominal aorta in the Control group (Figure 5A) was 1.86 ± 0.19 mm, the mean short-axis diameter of the local abdominal aorta in AAA-1w (Figure 5B) was 2.03 ± 0.29 mm, the mean local abdominal aorta in AAA-2w (Figure 5C) was 2.36 ± 0.24 mm, and the mean short-axis diameter in AAA-3w (Figure 5D) was 2.9 ± 0.05 mm, and the mean short-axis diameter of localized abdominal aorta in AAA-4w (Figure 5E) was 2.16 ± 0.03 mm. Figures 5F–I shows the long-axis diameter imaging of AAA-1w, AAA-2w, AAA-3w, and AAA-4w arteries, respectively. The diameter of the abdominal aorta can be measured from the transverse axis mode, and the trend of the abdominal aorta from normal to dilated can be seen from the long-axis mode. Consistent with the morphological results, the changes in diameter expansion of the abdominal aorta were better visualized by animal ultrasound imaging than by morphological observations, which showed the success of the AAA model as well as the dynamic development of the vascular expansion, as shown in Figure 5.

#### 3.2.3 Measurement of abdominal aortic structures

**FIGURE 6:**
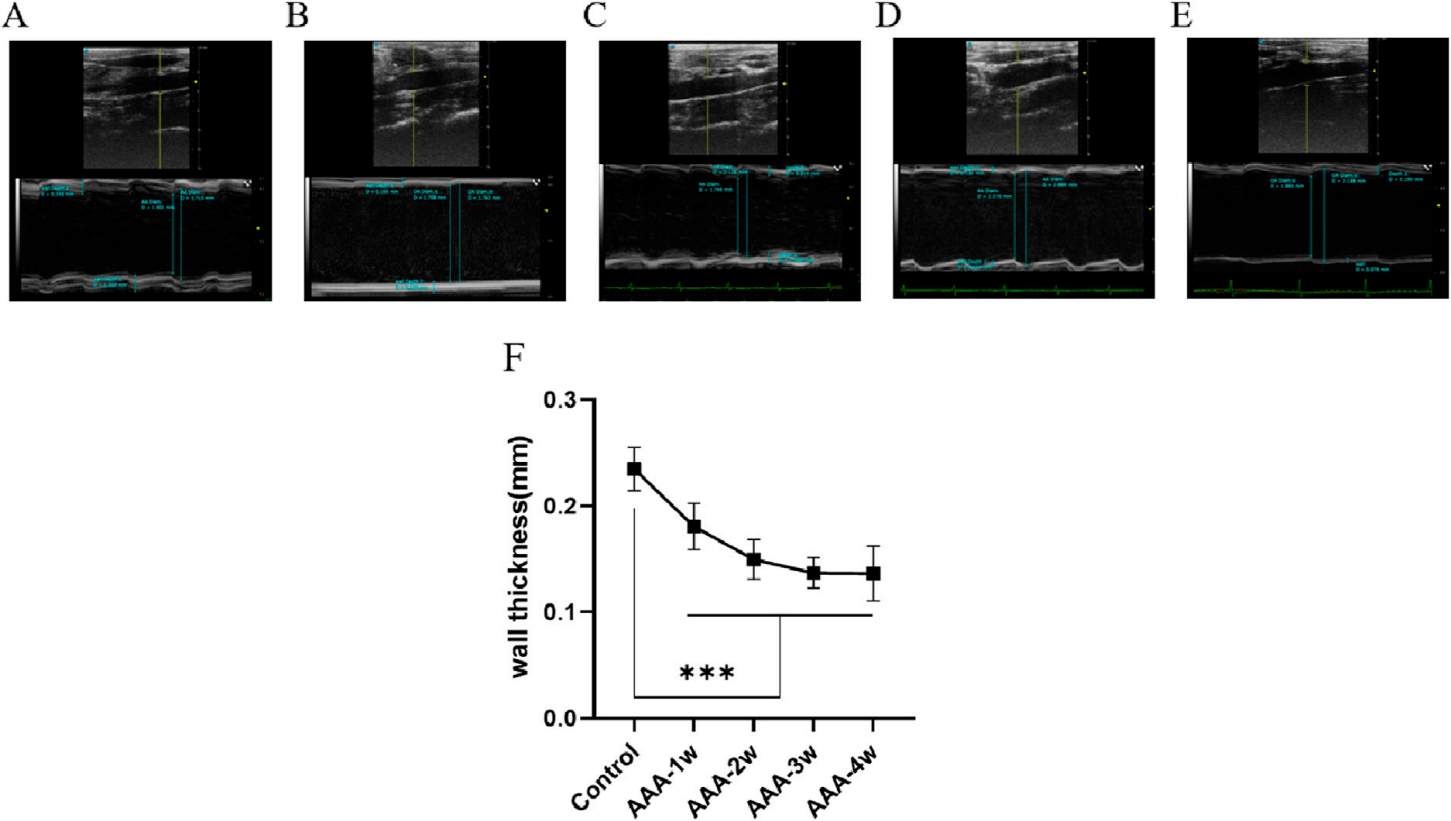
Abdominal aortic vessel wall thickness measurements. **(A)** Ultrasound images of the Control group. **(B**–**E)** are ultrasound images of t AAA-1w, AAA-2w, AAA-3w, and AAA-4w, respectively. **(F)** Structural maps for quantitative analysis of vessel wall thickness in each group. (^***^ *P* < 0.001 compared to Control group).

In the M-mode imaging mode, the vessel wall thickness was measured and is shown in Figure 6, where the vessel wall thickness in the AAA group became progressively thinner with the growth of AAA compared with the Control group (*P* < 0.001).

#### 3.2.4 Abdominal aortic blood flow measurements

**FIGURE 7:**
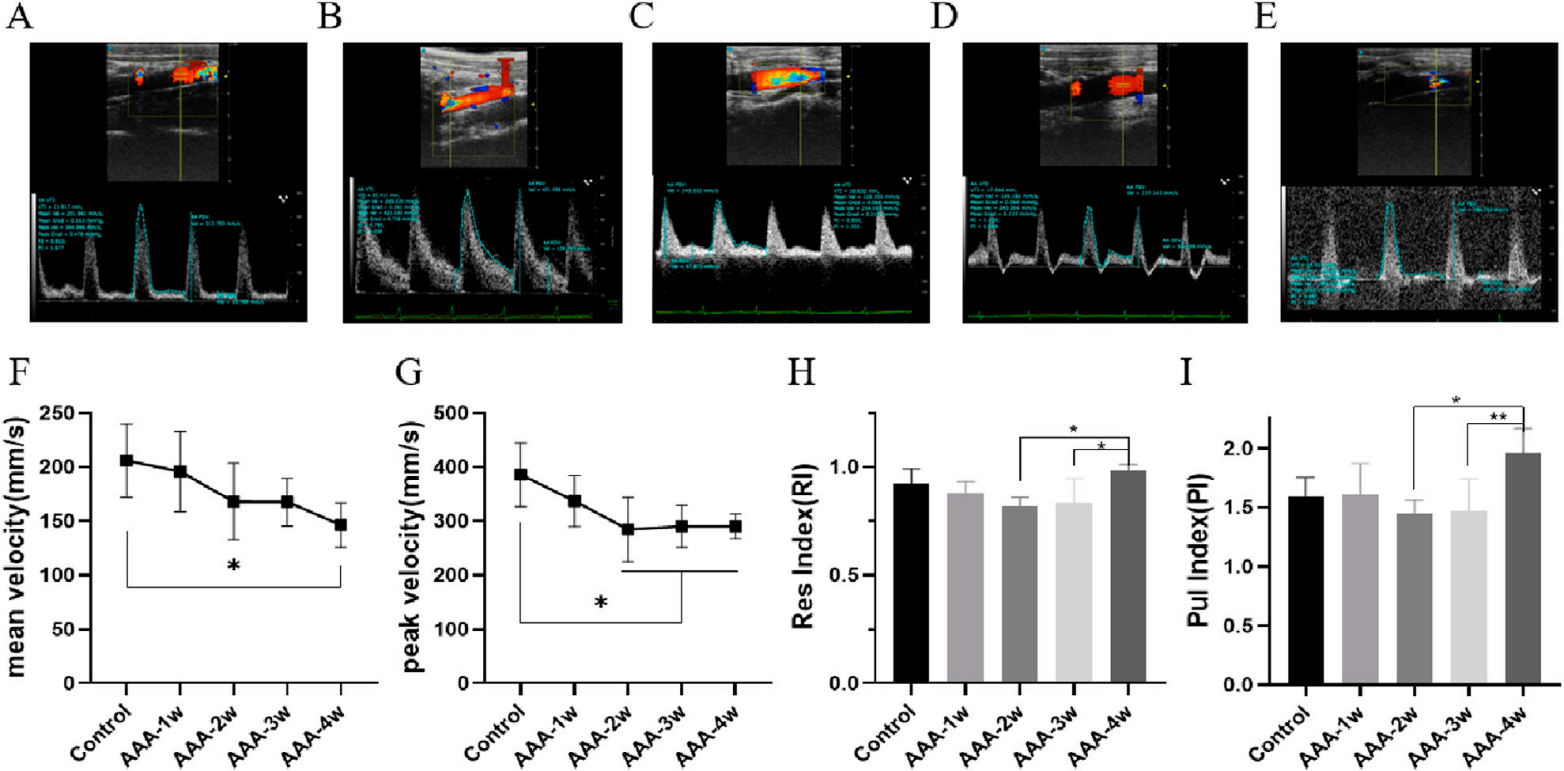
Hemodynamic profile of the abdominal aorta. **(A)** Control group pulsed Doppler images. **(B**–**E)** are pulsed Doppler images of AAA-1w, AAA-2w, AAA-3w, and AAA-4w, respectively. **(F)** Plot of the results of the quantitative analysis of the mean blood flow velocity in each group. **(G)** Plot of the results of quantitative analysis of peak blood flow velocity in each group. **(H)** Plot of quantitative analysis results of resistance indices for each group. **(I)** Plot of the results of the quantitative analysis of the beat indices of the groups. (^*^ *P* < 0.05, ^**^ *P* < 0.01 compared to Control group).

Hemodynamics was assessed using pulsed Doppler mode, which measured the mean flow velocity, peak flow velocity, resistance index, pulsatility index, per cardiac cycle. Compared with the Control group, the mean and peak blood flow velocities in the abdominal aorta of the AAA group were seen to decrease continuously with the progression of the aneurysm (*P* < 0.05), as shown in Figures 7F,G. RI and PI are ultrasound indexes for evaluating vascular resistance to blood flow, and there was no significant difference between the RI and PI of Control group and AAA groups, but with the expansion of arterial diameter there was a certain tendency for the resistance to blood flow to decrease but the difference was not significant. AAA-4w had a statistically significant difference from AAA-2w and AAA-3w (*P* < 0.05, *P* < 0.01) due to the reduced degree of diameter dilatation and increased resistance to blood flow, as shown in Figures 7H,I. Table 2 summarizes the ultrasound measurements of the abdominal aorta in the different groups.

**TABLE 2:** Comparison of diastolic diameter (Dd), wall thickness (wall thickness), mean flow velocity (Mean vel) peak flow velocity (Peak vel), resistance index (RI), and pulsatility index (PI) in different groups (Mean ± standard deviation).

	Control	AAA-1w	AAA-2w	AAA-3w	AAA-4w
Number	n = 4	n = 4	n = 4	n = 4	n = 4
Dd/mm	1.86 ±0.19	2.03 ±0.29	2.36 ±0.24	2.90 ±0.05	2.16 ±0.03
Wall thickness/mm	0.23 ±0.02	0.17 ±0.03	0.15 ±0.02	0.14 ±0.02	0.14 ±0.04
Mean velocity (mm/s)	205.95 ±21.51	195.93 ±72.66	168.10 ±51.10	167.72 ±2.73	146.53 ±20.45
Peak velocity (mm/s)	385.84 ±76.82	337.12 ±93.78	301.54 ±71.87	290.74 ±21.91	290.73 ±22.66
RI	0.92 ±0.07	0.88 ±0.05	0.82 ±0.04	0.83 ±0.11	0.98 ±0.03
PI	1.59 ±0.16	1.61 ±0.26	1.45 ±0.11	1.47 ±0.28	1.97 ±0.2

### 3.3 Vascular biomechanical analysis

#### 3.3.1 Comparison of vasoconstriction capacity

**FIGURE 8:**
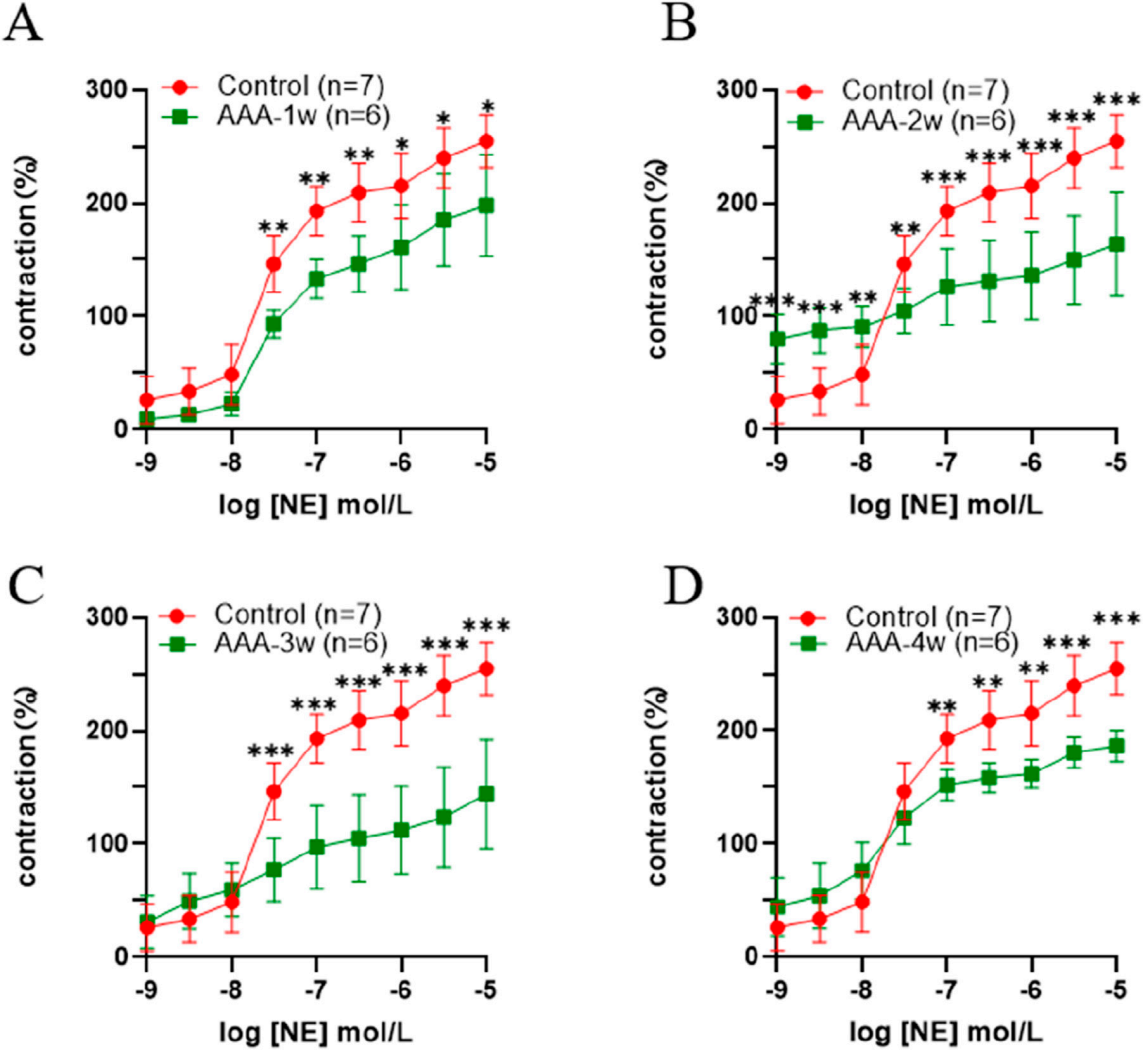
Active vasoconstriction of the abdominal aorta. **(A)** Comparison of contractility between Control group and AAA-1w. **(B)** Comparison of contractility between Control group and AAA-2w. **(C)** Comparison of contractility between Control group and AAA-3w. **(D)** Comparison of contractility of Control group with AAA-4w.

The AAA group exhibited a decreasing trend in concentration compared to the control group. As AAA advanced to 3 weeks, the concentration remained relatively stable at low levels, while it notably decreased at higher concentrations. The magnitude of contractility at the final concentration was analyzed, and compared with the Control group, there was a decrease in vasoconstriction in the AAA group from week one to week four vasoconstriction, with a 22% decrease (198.025 ± 86.669 vs. 254.871 ± 23.146) in vasoconstriction at AAA-1w (*P* < 0.05), a 36% decrease (163.908 ± 45.841 vs. 254.871 ± 23.146) at AAA-2w (*P* < 0.001), a 43% decrease (144.154 ± 47.933 vs. 254.871 ± 23.146) at AAA-3w (*P* < 0.001), and a AAA- 4w decreased (186.425 ± 13.709 vs. 254.871 ± 23.146) by 27% (*P* < 0.001), AAA-3w had the greatest decrease in contractility. The vascular contractility recovered slightly after reaching AAA-4w compared with AAA-2w and AAA-3w, indicating that the progression of AAA improved and recovery occurred by the fourth week of its development, which was in agreement with the previous morphological and ultrasonographic results, as shown in Figure 8.

#### 3.3.2 Analysis of vasoconstriction rate

See Table 3, which summarizes the values of K and K′ for each group at different concentrations (K is the slope of the *Ts-Tm* segment and K′ is the slope of the *Tm-Te* segment), and compares the rates of K *versus* K′ within each group. There was a significant difference between K values and K′ in the Control group at all concentrations, and for the Control group, the addition of different concentrations of the contractile drug reached *Tm* at a fast rate of contraction. For AAA-1w, there was a significant difference between K values and K' (0.0171 ± 0.0029 vs. 0.0038 ± 0.0043) at concentrations of 10^–7.5^, for AAA-2w, there was a significant difference between K values at 10^–9^ concentrations and K' (0.0201 ± 0.0108 vs. 0.0028 ± 0.0013) for AAA-3w, there was a significant difference between K values and K′ at 10^–8.5^, 10^−7^concentrations, and for AAA-4w, there was a significant difference between K values and K′ at 10^–9^, 10^–8.5^, 10^–8^, 10^–7.5^, 10^–5.5^, 10^–5^ concentrations. Overall, the K values of the different cycles were greater than K′, suggesting that the overall tendency of the addition of the different contractile agents was that the blood vessels showed a first fast and then slow contraction phenomenon.

**TABLE 3:** Comparison of contraction rates (Mean ± standard deviation) at different concentrations in each group.

		Control	AAA-1w	AAA-2w	AAA-3w	AAA-4w
10^–9^	K_1_	0.0256 ± 0.0014	0.0012 ± 0.0011	0.0201 ± 0.0108	0.0107 ± 0.005	0.0165 ± 0.0031
	K_1_'	0.0021 ± 0.0004	0.0009 ± 0.001	0.0028 ± 0.0013	0.0049 ± 0.01	0.0029 ± 0.0025
P value K_1_-K_1_'		****	ns	****	ns	***
10^–8.5^	K_2_	0.0034 ± 0	0.0005 ± 0.0001	0.0005 ± 0.0001	0.0024 ± 0.0002	0.0029 ± 0
	K_2_'	0.0005 ± 0.0002	0.0004 ± 0.0004	0.0003 ± 0.0003	0.0015 ± 0.0001	0.0014 ± 0.0006
P value K_2_-K_2_'		****	ns	ns	**	****
10^–8^	K_3_	00,092 ± 0.0049	0.0025 ± 0	0.0007 ± 0.0009	0.0022 ± 0.0006	0.0072 ± 0.0021
	K_3_'	0.0007 ± 0.0007	0.0003 ± 0.0002	0.0005 ± 0.0003	0.0009 ± 0.0018	0.0016 ± 0.0006
P value K_3_-K_3_'		****	ns	ns	ns	***
10^–7.5^	K_4_	0.0452 ± 0.0034	0.0171 ± 0.0029	0.0018 ± 0.0003	0.0034 ± 0.0018	0.0156 ± 0.0034
	K_4_'	0.0057 ± 0.0049	0.0038 ± 0.0043	0.001 ± 0	0.0012 ± 0.0011	0.0022 ± 0.0009
P value K_4_-K_4_'		****	**	ns	ns	***
10^–7^	K_5_	0.1683 ± 0.0212	0.0133 ± 0.0018	0.0242 ± 0.0431	0.0363 ± 0.0221	0.0116 ± 0.0016
	K_5_'	0.0211 ± 0.0206	0.0024 ± 0.0035	0.0011 ± 0.0001	0.0012 ± 0.0004	0.0012 ± 0.0007
P value K_5_-K_5_'		****	ns	ns	***	ns
10^–6.5^	K_6_	0.0049 ± 0.0004	0.0023 ± 0.0005	0.0006 ± 0	0.0013 ± 0.0003	0.0011 ± 0.0002
	K_6_'	0.0005 ± 0.0002	0.0013 ± 0.0004	0.0001 ± 0.0002	0.0005 ± 0	0.0008 ± 0.0008
P value K_6_-K_6_'		****	ns	ns	ns	ns
10^–6^	K_7_	0.0058 ± 0.0007	0.0017 ± 0.0013	0.0005 ± 0.0004	0.0009 ± 0.0003	0.0006 ± 0
	K_7_'	0.0013 ± 0.0001	0.001 ± 0.0021	0.0002 ± 0.0003	0.0008 ± 0.0001	0.0004 ± 0.0001
P value K_7_-K_7_'		****	ns	ns	ns	ns
10^–5.5^	K_8_	0.0149 ± 0.0016	0.0044 ± 0.0018	0.0035 ± 0.001	0.0032 ± 0.0008	0.0054 ± 0.0003
	K_8_'	0.0022 ± 0.0021	0.0021 ± 0.0028	0.0008 ± 0.0001	0.0005 ± 0	0.0011 ± 0.0006
P value K_8_-K_8_'		****	ns	ns	ns	***
10^–5^	K_9_	0.0085 ± 0.0017	0.0026 ± 0.0045	0.0026 ± 0.0001	0.0031 ± 0.0013	0.0052 ± 0.0014
	K_9_'	0.0003 ± 0.0002	0.0013 ± 0.0006	0.0006 ± 0.0001	0.0022 ± 0.0025	0.001 ± 0.0001
P value K_9_-K_9_'		****	ns	ns	ns	**

(^**^ *P* < 0.01,^***^ *P* < 0.001,^****^ *P* < 0.0001).

#### 3.3.3 Vascular contraction stress analysis

**FIGURE 9:**
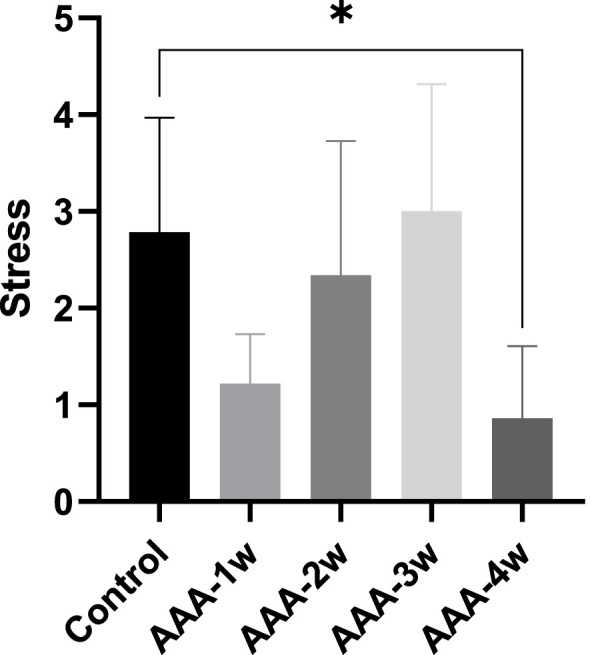
Vascular contraction stress analysis: The AAA-1w group, AAA-2w group, AAA-3w group, and AAA-4w group were compared with the control group, with *P < 0.05 indicating statistical significance.

The vascular contractile stress at the final concentration was calculated for each group. It is observed that the contractile stress in the vessels of the AAA group decreased compared to the control group in week 1 (1.218 ± 0.513 vs. 2.787 ± 1.183). Subsequently, the vascular contractile stress gradually increased. By the fourth week of the recovery phase, the contractile stress measured 0.860 ± 0.748, as depicted in Figure 9.

### 3.4 Pathological and histological analysis

#### 3.4.1 HE staining of pathology sections

**FIGURE 10:**
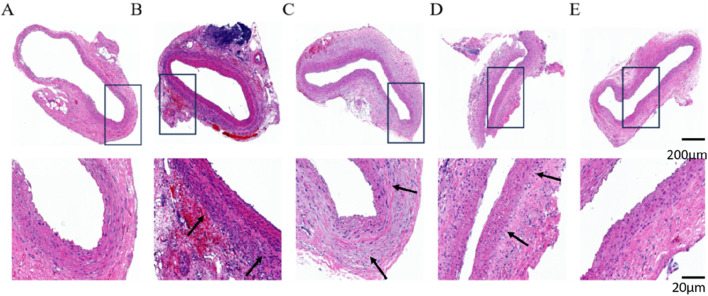
HE staining of rat abdominal aorta, black arrows indicate vascular inflammatory infiltration, increased inflammatory cells. **(A)** HE staining of abdominal aorta in Control group. **(B)** AAA-1w HE staining of the abdominal aorta. **(C)** AAA-2w HE staining of the abdominal aorta. **(D)** AAA-3w HE staining of the abdominal aorta. **(E)** AAA-4w HE staining of the abdominal aorta. Bar: 200 μm for the first row, 20 μm for the second row.

HE staining results showed that the abdominal aortic wall in the Control group had a normal structure, with a uniform distribution of intima-media, neatly arranged, and no obvious inflammatory cell infiltration. Compared with the Control group, AAA-1w, AAA-2w and AAA-3w showed that the normal structure of the abdominal aortic wall was damaged, and the mesangial cells of the blood vessels were unevenly distributed and disordered in the order of arrangement, accompanied by obvious inflammatory cell infiltration in the local area as shown by the black arrows, in contrast, the cells were more uniformly distributed and relatively neatly arranged with less inflammatory cell infiltration in AAA-4w (Figure 10).

#### 3.4.2 Masson staining of pathologic sections

**FIGURE 11:**
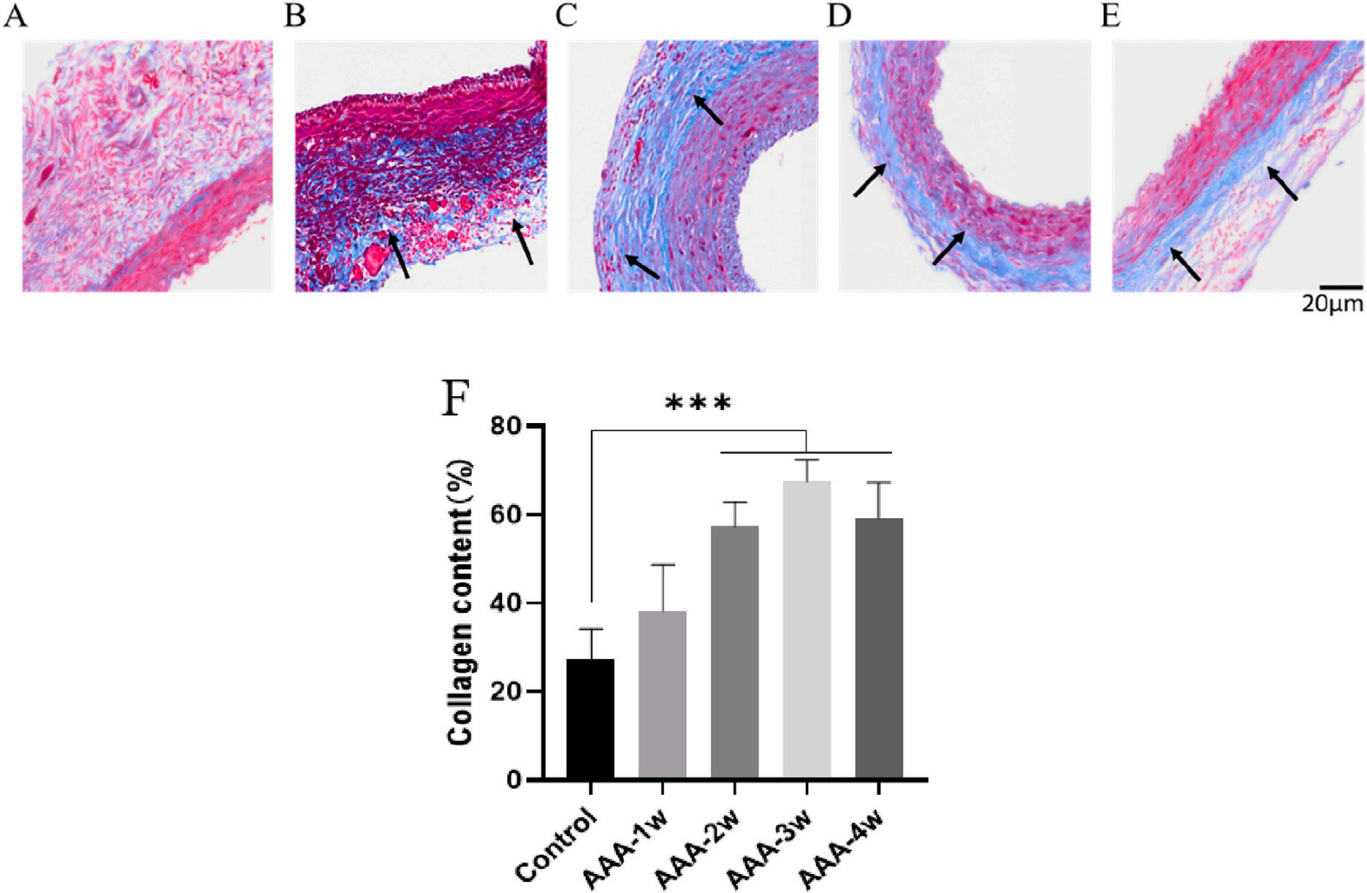
Masson staining of rat abdominal aorta, black arrows indicate vascular fibrosis. **(A)** Masson staining of abdominal aorta in Control group. **(B)** AAA-1w Masson staining of the abdominal aorta. **(C)** AAA-2w Masson staining of the abdominal aorta. **(D)** AAA-3w Masson staining of the abdominal aorta. **(E)** AAA-4w Masson staining of the abdominal aorta. **(F)** Graph of quantitative analysis results of Masson staining (^***^ *P* < 0.001 compared to Control group). Bar: 20 μm.

Degradation of extracellular matrix and metabolic imbalance is one of the important mechanisms in the development of actinic tumors and can further stimulate compensatory deposition of collagen fibers and cause metabolic imbalance of collagen fibers. To assess vascular collagen deposition, we performed Masson staining to analyze the degree of vascular fibrosis, and the black arrows indicated vascular fibrosis. As shown in Figure 11, the results showed that vascular fibrosis became progressively more severe with the progression of AAA, and the degree of fibrosis stabilized at week 4 (*P* < 0.001).

#### 3.4.3 EVG-modified weigert method staining of pathology sections

**FIGURE 12:**
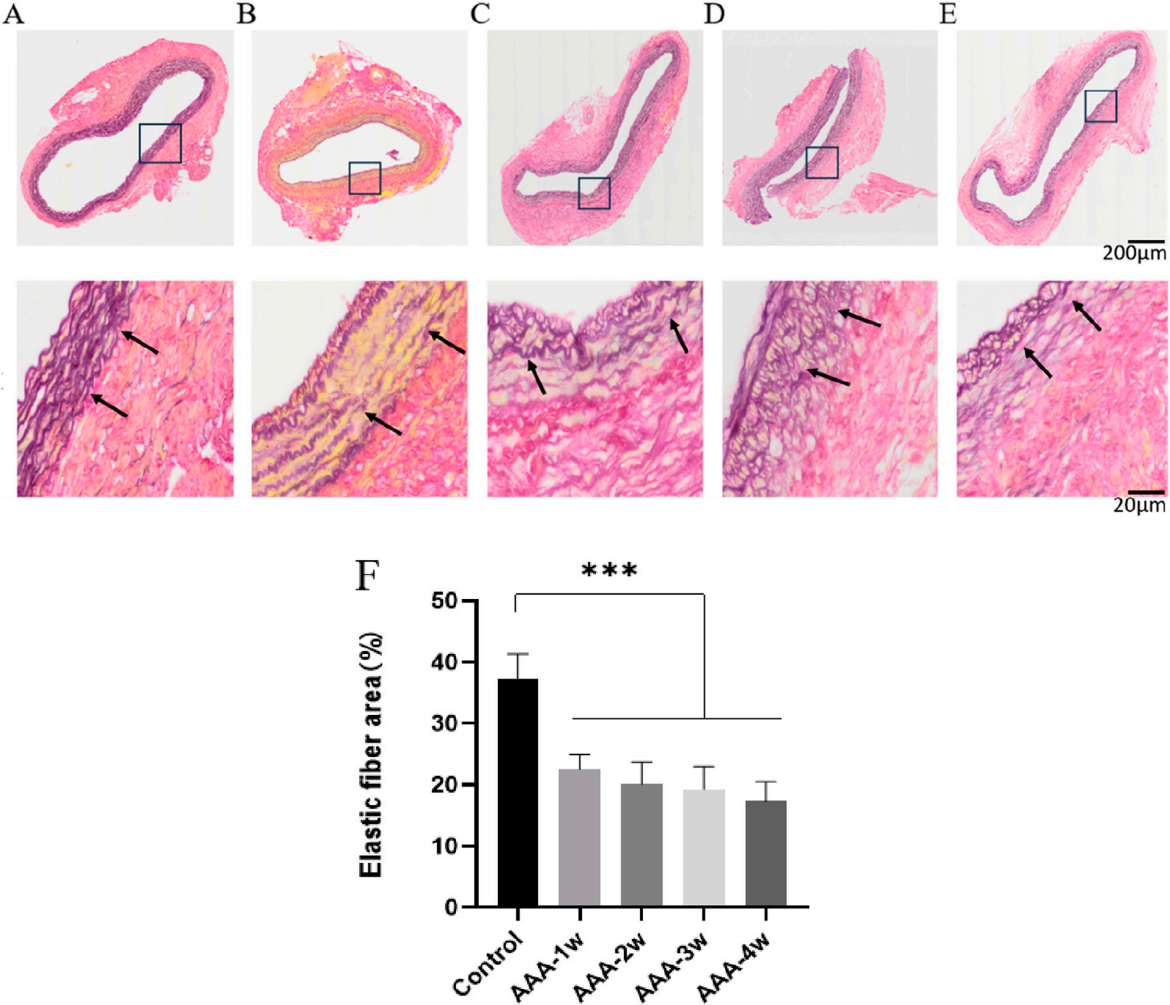
EVG staining of rat abdominal aorta, black arrows indicate elastic fibers and broken elastic fibers. **(A)** EVG staining of abdominal aorta in Control group. **(B)** AAA-1w EVG staining of the abdominal aorta. **(C)** AAA-2w EVG staining of the abdominal aorta. **(D)** AAA-3w EVG staining of the abdominal aorta. **(E)** AAA-4w EVG staining of the abdominal aorta. **(F)** Graph of quantitative analysis results of EVG staining (^***^ *P* < 0.001 compared to Control group). Bar: 200 μm for the first row, 20 μm for the second row.

Degradation of elastic fibers is the initiating step in the formation of aneurysmal dilatation of the artery, which in turn allows the aneurysm body to continue to expand its to rupture. In order to observe the effect of elastic fiber degradation on the aneurysm formation process with the extension of time, we adopted the EVG-modified Weigert method to observe the effect of elastic fibers in the vessel wall. The results showed that the elastic fibers in the abdominal aorta of rats in Control group were wavy, parallel, dense and uniformly arranged in the intima media of the blood vessels, which were arranged in a more regular manner and had better integrity, with complete elastic fibers indicated by black arrows. Elastic fibers of the AAA group underwent degradation of varying degrees, and their morphology appeared to be altered. Staining results showed that the morphology of the elastic fibers in the vessel wall of the AAA group was flattened, the wavy structure disappeared, the arrangement was sparse and disorganized, and some of them were fractured, and the degradation of the elastic fibers was very significant (*P* < 0.001), and the black arrows indicated the fractured elastic fibers in the figure (Figure 12).

#### 3.4.4 Immunofluorescence staining of pathology sections

**FIGURE 13:**
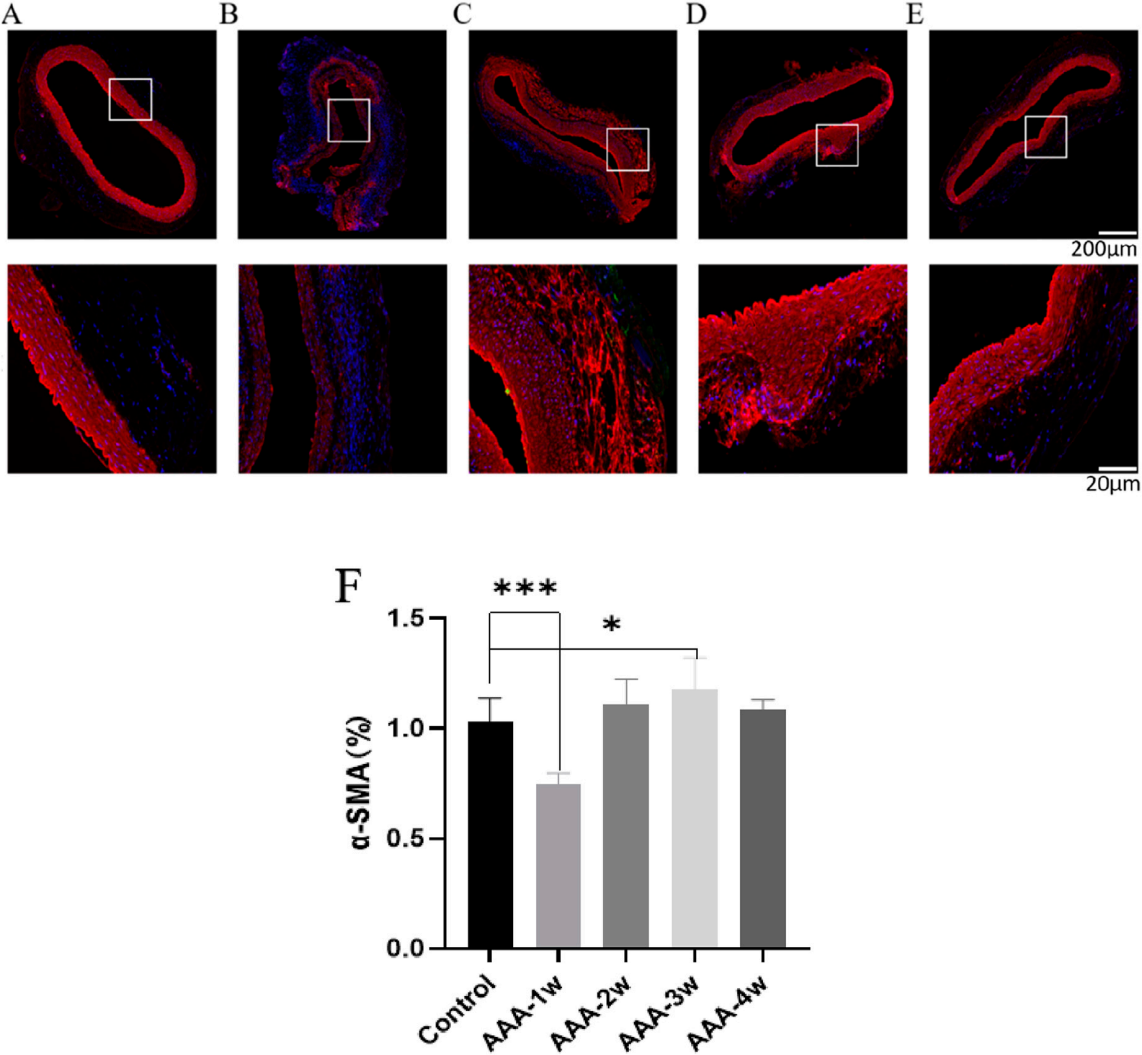
α-SMA staining of rat abdominal aorta. **(A)** α-SMA staining of abdominal aorta in Control group. **(B)** AAA-1w α-SMA staining of the abdominal aorta. **(C)** AAA-2w α-SMA staining of the abdominal aorta. **(D)** AAA-3w α-SMA staining of the abdominal aorta. **(E)** AAA-4w α-SMA staining of the abdominal aorta. **(F)** Graph of quantitative analysis results of α-SMA staining (^*^ *P* < 0.05, ^***^ *P* < 0.001 compared to Control group). Bar: 200 μm for the first row, 20 μm for the second row.

The smooth muscle cells in Control group were regular in morphology and uniformly arranged in the middle layer of blood vessels, AAA-1w vascular smooth muscle cells were obviously destroyed, only some smooth muscle cells remained, and their content was significantly lower than that of Control group (*P* < 0.001), thereafter, the smooth muscle cell content gradually increased, and the growth of smooth muscle cell content tended to be leveled off at 4 weeks postoperatively (Figure 13).

## 4 Discussion

This study primarily explores the changes in the mechanical properties and structural components during the development of AAA. The research highlights significant changes in arterial contractility and compositional alterations during vascular lesion progression. The study arrived at the following conclusions: (1) A repairable AAA model in rats can be established through the external application of porcine pancreatic elastase; (2) The vasoconstriction capacity shows a gradual decrease with the development of AAA; (3) Contraction stress notably decreases in the early stages of AAA, with stress levels rising over time and eventually stabilizing.

The arterial wall can be considered a composite material composed of collagen fibers and smooth muscle cells embedded in an elastic matrix. As AAA severity progresses from one to 3 weeks, arterial contractility gradually decreases, primarily due to increased inflammation and the rupture of collagen and elastic fibers, as evidenced by histological staining. Arterial elasticity is mainly expressed through the active contraction of smooth muscle cells and the passive contraction of the elastic layer, which consists of collagen and elastic fibers. Smooth muscle cell contraction regulates vascular diameter, blood pressure, and blood flow distribution (Cao et al., 2022). Dysfunction in smooth muscle cells affects the active dynamic changes in blood vessels. The passive properties of blood vessels largely depend on the extracellular matrix (ECM) network, including elastin and collagen, which are the main structural proteins of the arterial wall. Intact elastin is crucial for maintaining the normal shape and compliance of the vessel wall. At low strain, elastin dominates, making the vessel wall relatively flexible; at high strain, the elastic modulus of collagen becomes dominant, providing tensile strength to the vessel (Vorp, 2007; Lu et al., 2021). By the fourth week, the active components of the arterial wall must continuously regenerate and remodel to maintain systemic integrity and function, leading to a recovery in active contractility. Remodeling of structural proteins has been shown to play a significant role in the mechanical behavior of the vessel wall during AAA development (Vorp, 2007).

In this study, we monitored the biomechanical changes occurring during AAA development and remodeling using small animal ultrasound. We found that the loss of aortic structure leads to weakened vascular function and aortic dilation, resulting in decreased mean and peak blood flow velocities in AAA. Interestingly, as AAA progressed to the fourth week, the extent of vascular dilation and contraction reduction decreased, which may indicate a self-repair phenomenon during vascular remodeling. This can be explained by the law of Thomas, which states that when blood flow changes, the initial response is contraction or relaxation of smooth muscle cells to alter the arterial wall diameter, possibly signaled by endothelial cells sensing changes in shear stress. Over time, smooth muscle cells remodel themselves and the ECM to increase or decrease arterial diameter, adapting to new volume flow and maintaining constant shear stress (Dobrin, 1989; Vorp, 2007). Although thinner vessel walls may experience higher local stress, some studies suggest that aneurysm rupture does not correlate well with vessel wall thickness (Sugita and Matsumoto, 2018).

Stress is defined as the force applied to an object divided by the surface area over which the force acts. Stress on the vessel wall includes shear stress from blood flow within the lumen, longitudinal stress from surrounding tissues, and circumferential stress from blood pressure. For materials like vessel walls, the relationship between stress and strain is nonlinear; increased strain leads to increased stress, and *vice versa* (Wagenseil and Mecham, 2009). AAA rupture occurs when the stress applied exceeds the wall strength. In this study, contractile stress initially decreased rapidly during AAA progression, then gradually increased. By the fourth week, the decrease in contractile stress was consistent with ultrasound and active vascular contraction results, suggesting self-healing of AAA at this stage.

The development and rupture of AAA result from the interplay of biological and biomechanical changes, leading to ECM remodeling and degradation. These changes result from the biomechanical stress transmission between cells and the ECM. Wall stress analysis is more sensitive and specific in predicting AAA rupture and can identify AAAs that can be safely observed. Elevated wall stress can be detected before rupture occurs, allowing time for intervention. Remodeling alters the direction and composition of the AAA wall components. Consequently, AAA tissue is significantly weaker than normal aorta, and due to compensatory remodeling processes, AAA stiffness initially appears to increase. As this adaptive process begins to fail, stiffness decreases and compliance reduces (Annambhotla et al., 2008; Rodríguez et al., 2008). Minimally invasive stent graft placement can isolate the degraded vessel wall, preventing further AAA expansion and rupture, but there is currently a lack of quantitative standards for assessing post-operative endovascular repair effectiveness (Petsophonsakul et al., 2019; Zhao et al., 2021). This study proposes a clear biomechanical framework for evaluating AAA development.

Histologically, we observed inflammatory infiltration, collagen fiber deposition, elastic fiber rupture, and a reduction in mesenteric smooth muscle cells within the aneurysm wall, consistent with the findings of Annambhotla (Annambhotla et al., 2008). However, data regarding changes in collagen are contradictory; some authors report an increase in collagen fraction (Rizzo et al., 1989), while others report a decrease or no change (Friedmann et al., 1992; Carmo et al., 2002). In our experiment, we observed a gradual deposition of collagen as AAA progressed to the third week, aligning with the inflammatory infiltration seen in HE-stained AAA, and a slight reduction in inflammation by the fourth week, consistent with observations by Price (Police et al., 2009). Vascular tissue growth occurs in a balanced environment, which is disrupted during AAA development. Initially, an inflammatory environment with the release of numerous inflammatory factors leads to vascular dilation and continuous degradation of elastic fibers, which tend to deteriorate with age. Fibroblasts activate synthesis mechanisms, increasing collagen fiber content, ultimately transferring the load from elastin to unloaded collagen. Collagen maintains mechanical equilibrium during tissue remodeling, supporting arteries and providing rigidity to the arterial wall, which may explain the reduced vascular dilation and contraction observed by the fourth week. If collagen also has mechanical defects, is abnormal, and is susceptible to enzymatic degradation, it becomes more prone to degradation in the vessel wall, making the vessel unable to withstand the luminal pressure that generates tensile force, eventually leading to rupture (Dobrin, 1989).

### 4.1 Limitations

Firstly, the growth and remodeling period studied was relatively short, focusing solely on the vascular state at 1, 2, 3, and 4 weeks post-modeling of AAA. Secondly, there was no discussion on the mechanical strength of the blood vessels, as the vessel strength might undergo alterations after drug administration, and testing these two mechanical parameters cannot be completed through a single system. Thirdly, due to the necessity of euthanizing animals every week for experimentation, there was no increase in the number of animals after obtaining statistically significant results. Fourthly, the study concentrated exclusively on biomechanical changes during the vascular growth and remodeling process of AAA, without delving deeper into the underlying biological mechanisms that influence these mechanical alterations.

## 5 Conclusion

The external application of porcine pancreatic elastase can establish a modifiable AAA model, wherein the inflammatory response can be exacerbated or ameliorated. Throughout the progression of AAA, parameters such as vascular diameter, vascular wall thickness, blood flow velocity, and vascular contractility exhibit a gradual decline. Pathological staining demonstrates a close association between contractility and the presence of vascular elastic fibers and collagen fibers. By the fourth week of AAA development, there is a slight reduction in inflammation, gradual deposition of tissue collagen, a decrease in smooth muscle cell proliferation, and a notable increase in active contractility. This sheds light on the intricate relationship between active vascular contractility and vascular composition.

## Data Availability

The original contributions presented in the study are included in the article/supplementary material, further inquiries can be directed to the corresponding authors.

## References

[B1] Annambhotla S. Bourgeois S. Wang X. Lin P. H. Yao Q. Chen C. (2008). Recent advances in molecular mechanisms of abdominal aortic aneurysm formation. World J. Surg. 32, 976–986. 10.1007/s00268-007-9456-x 18259804 PMC2927355

[B2] Cao G. Xuan X. Hu J. Zhang R. Jin H. Dong H. (2022). How vascular smooth muscle cell phenotype switching contributes to vascular disease. Cell Commun. Signal 20, 180. 10.1186/s12964-022-00993-2 36411459 PMC9677683

[B3] Carmo M. Colombo L. Bruno A. Corsi F. R. Roncoroni L. Cuttin M. S. (2002). Alteration of elastin, collagen and their cross-links in abdominal aortic aneurysms. Eur. J. Vasc. Endovasc. Surg. 23, 543–549. 10.1053/ejvs.2002.1620 12093072

[B4] Chen C. Wang Y. Cao Y. Wang Q. Anwaier G. Zhang Q. (2020). Mechanisms underlying the inhibitory effects of probucol on elastase-induced abdominal aortic aneurysm in mice. Br. J. Pharmacol. 177, 204–216. 10.1111/bph.14857 31478560 PMC6976779

[B5] Chen W. Tumanov S. Stanley C. P. Kong S. M. Y. Nadel J. Vigder N. (2023). Destabilization of atherosclerotic plaque by bilirubin deficiency. Circ. Res. 132, 812–827. 10.1161/circresaha.122.322418 36876485

[B6] Cheng J. K. Wagenseil J. E. (2012). Extracellular matrix and the mechanics of large artery development. Biomech. Model Mechanobiol. 11, 1169–1186. 10.1007/s10237-012-0405-8 22584609 PMC3463721

[B7] Cho M. J. Lee M. R. Park J. G. (2023). Aortic aneurysms: current pathogenesis and therapeutic targets. Exp. Mol. Med. 55, 2519–2530. 10.1038/s12276-023-01130-w 38036736 PMC10766996

[B8] Cullen J. M. Lu G. Shannon A. H. Su G. Sharma A. Salmon M. (2019). A novel swine model of abdominal aortic aneurysm. J. Vasc. Surg. 70, 252–260.e2. 10.1016/j.jvs.2018.09.057 30591288 PMC6591111

[B9] Davis F. M. Rateri D. L. Daugherty A. (2015). Abdominal aortic aneurysm: novel mechanisms and therapies. Curr. Opin. Cardiol. 30, 566–573. 10.1097/hco.0000000000000216 26352243 PMC4624089

[B10] Del Campo L. Ferrer M. (2015). Wire myography to study vascular tone and vascular structure of isolated mouse arteries. Methods Mol. Biol. 1339, 255–276. 10.1007/978-1-4939-2929-0_18 26445795

[B11] Dobrin P. B. (1989). Pathophysiology and pathogenesis of aortic aneurysms. Current concepts. Surg. Clin. North Am. 69, 687–703. 10.1016/s0039-6109(16)44876-0 2665139

[B12] Friedmann P. W. Baxter B. T. Mcgee G. S. Shively V. P. Drummond I. a.S. Dixit S. N. (1992). Elastin content, cross-links, and mRNA in normal and aneurysmal human aorta. J. Vasc. Surg. 16, 0192–0200. 10.1067/mva.1992.36429 1495142

[B13] Gao J. Cao H. Hu G. Wu Y. Xu Y. Cui H. (2023). The mechanism and therapy of aortic aneurysms. Signal Transduct. Target Ther. 8, 55. 10.1038/s41392-023-01325-7 36737432 PMC9898314

[B14] Kandail H. Hamady M. Xu X. Y. (2014). Patient-specific analysis of displacement forces acting on fenestrated stent grafts for endovascular aneurysm repair. J. Biomechanics 47, 3546–3554. 10.1016/j.jbiomech.2014.08.011 25267572

[B15] Krishnan J. Hennen E. M. Ao M. Kirabo A. Ahmad T. De La Visitacion N. (2024). NETosis drives blood pressure elevation and vascular dysfunction in hypertension. Circ. Res. 134, 1483–1494. 10.1161/circresaha.123.323897 38666386 PMC11116040

[B16] Li Z. Zhao Z. Cai Z. Sun Y. Li L. Yao F. (2020). Runx2 (Runt-related transcription factor 2)-mediated microcalcification is a novel pathological characteristic and potential mediator of abdominal aortic aneurysm. Arterioscler. Thromb. Vasc. Biol. 40, 1352–1369. 10.1161/atvbaha.119.314113 32212850

[B17] Lu H. Du W. Ren L. Hamblin M. H. Becker R. C. Chen Y. E. (2021). Vascular smooth muscle cells in aortic aneurysm: from genetics to mechanisms. J. Am. Heart Assoc. 10, e023601. 10.1161/jaha.121.023601 34796717 PMC9075263

[B18] Lu Y. Wu H. Li J. Gong Y. Ma J. Kassab G. S. (2017). Passive and active triaxial wall mechanics in a two-layer model of porcine coronary artery. Sci. Rep. 7, 13911. 10.1038/s41598-017-14276-1 29066847 PMC5655692

[B19] Ma X. Li Y. F. Gao Q. Ye Z. G. Lu X. J. Wang H. P. (2008). Inhibition of superoxide anion-mediated impairment of endothelium by treatment with luteolin and apigenin in rat mesenteric artery. Life Sci. 83, 110–117. 10.1016/j.lfs.2008.05.010 18558413

[B20] Malkawi A. H. Hinchliffe R. J. Xu Y. Holt P. J. Loftus I. M. Thompson M. M. (2010). Patient-specific biomechanical profiling in abdominal aortic aneurysm development and rupture. J. Vasc. Surg. 52, 480–488. 10.1016/j.jvs.2010.01.029 20395107

[B21] Melin L. G. Dall J. H. Lindholt J. S. Steffensen L. B. Beck H. C. Elkrog S. L. (2022). Cycloastragenol inhibits experimental abdominal aortic aneurysm progression. Biomedicines 10, 359. 10.3390/biomedicines10020359 35203568 PMC8962318

[B22] Morris S. T. Mcmurray J. J. Spiers A. Jardine A. G. (2001). Impaired endothelial function in isolated human uremic resistance arteries. Kidney Int. 60, 1077–1082. 10.1046/j.1523-1755.2001.0600031077.x 11532102

[B23] Niestrawska J. A. Regitnig P. Viertler C. Cohnert T. U. Babu A. R. Holzapfel G. A. (2019). The role of tissue remodeling in mechanics and pathogenesis of abdominal aortic aneurysms. Acta Biomater. 88, 149–161. 10.1016/j.actbio.2019.01.070 30735809

[B24] Petsophonsakul P. Furmanik M. Forsythe R. Dweck M. Schurink G. W. Natour E. (2019). Role of vascular smooth muscle cell phenotypic switching and calcification in aortic aneurysm formation. Arterioscler. Thromb. Vasc. Biol. 39, 1351–1368. 10.1161/atvbaha.119.312787 31144989

[B25] Phillips E. H. Yrineo A. A. Schroeder H. D. Wilson K. E. Cheng J. X. Goergen C. J. (2015). Morphological and biomechanical differences in the elastase and AngII apoE(-/-) rodent models of abdominal aortic aneurysms. Biomed. Res. Int. 2015, 413189. 10.1155/2015/413189 26064906 PMC4433642

[B26] Pinard A. Jones G. T. Milewicz D. M. (2019). Genetics of thoracic and abdominal aortic diseases. Circ. Res. 124, 588–606. 10.1161/circresaha.118.312436 30763214 PMC6428422

[B27] Police S. B. Thatcher S. E. Charnigo R. Daugherty A. Cassis L. A. (2009). Obesity promotes inflammation in periaortic adipose tissue and angiotensin II-induced abdominal aortic aneurysm formation. Arterioscler. Thromb. Vasc. Biol. 29, 1458–1464. 10.1161/atvbaha.109.192658 19608970 PMC2753598

[B28] Qian G. Adeyanju O. Olajuyin A. Guo X. (2022). Abdominal aortic aneurysm formation with a focus on vascular smooth muscle cells. Life (Basel) 12, 191. 10.3390/life12020191 35207478 PMC8880357

[B29] Rizzo R. J. Mccarthy W. J. Dixit S. N. Lilly M. P. Shively V. P. Flinn W. R. (1989). Collagen types and matrix protein content in human abdominal aortic aneurysms. J. Vasc. Surg. 10, 365–373. 10.1016/0741-5214(89)90409-6 2795760

[B30] Rodríguez J. F. Ruiz C. Doblaré M. Holzapfel G. A. (2008). Mechanical stresses in abdominal aortic aneurysms: influence of diameter, asymmetry, and material anisotropy. J. Biomech. Eng. 130, 021023. 10.1115/1.2898830 18412510

[B31] Romary D. J. Berman A. G. Goergen C. J. (2019). High-frequency murine ultrasound provides enhanced metrics of BAPN-induced AAA growth. Am. J. Physiol. Heart Circ. Physiol. 317, H981–h990. 10.1152/ajpheart.00300.2019 31559828 PMC6879923

[B32] Sharma N. Dev R. Belenchia A. M. Aroor A. R. Whaley-Connell A. Pulakat L. (2019). Deficiency of IL12p40 (Interleukin 12 p40) promotes ang II (Angiotensin II)-Induced abdominal aortic aneurysm. Arterioscler. Thromb. Vasc. Biol. 39, 212–223. 10.1161/atvbaha.118.311969 30580570 PMC6355331

[B33] Sommer G. Sherifova S. Oberwalder P. J. Dapunt O. E. Ursomanno P. A. Deanda A. (2016). Mechanical strength of aneurysmatic and dissected human thoracic aortas at different shear loading modes. J. Biomech. 49, 2374–2382. 10.1016/j.jbiomech.2016.02.042 26970889 PMC5435125

[B34] Sugita S. Matsumoto T. (2018). Local distribution of collagen fibers determines crack initiation site and its propagation direction during aortic rupture. Biomechanics Model. Mechanobiol. 17, 577–587. 10.1007/s10237-017-0979-2 29134291

[B35] Tran V. Brettle H. Diep H. Dinh Q. N. O'keeffe M. Fanson K. V. (2023). Sex-specific effects of a high fat diet on aortic inflammation and dysfunction. Sci. Rep. 13, 21644. 10.1038/s41598-023-47903-1 38062083 PMC10703842

[B36] Tyerman Z. Dahl J. Shannon A. Johnston W. F. Pope N. H. Lu G. (2019). Murine surgical model of topical elastase induced descending thoracic aortic aneurysm. J. Vis. Exp. 10.3791/60105 PMC11029371 31498329

[B37] Vorp D. A. (2007). Biomechanics of abdominal aortic aneurysm. J. Biomech. 40, 1887–1902. 10.1016/j.jbiomech.2006.09.003 17254589 PMC2692528

[B38] Wagenseil J. E. Mecham R. P. (2009). Vascular extracellular matrix and arterial mechanics. Physiol. Rev. 89, 957–989. 10.1152/physrev.00041.2008 19584318 PMC2775470

[B39] Xue C. Zhao G. Zhao Y. Chen Y. E. Zhang J. (2022). Mouse abdominal aortic aneurysm model induced by perivascular application of elastase. J. Vis. Exp. 10.3791/63608 PMC9450023 35225256

[B40] Zhang F. Li K. Zhang W. Zhao Z. Chang F. Du J. (2024). Ganglioside GM3 protects against abdominal aortic aneurysm by suppressing ferroptosis. Circulation 149, 843–859. 10.1161/circulationaha.123.066110 38018467

[B41] Zhao D. Niu P. Sun X. Yin Z. Tan W. Huo Y. (2020). Mechanical difference of left ventricle between rabbits of myocardial infarction and hypertrophy. J. Biomechanics 111, 110021. 10.1016/j.jbiomech.2020.110021 32927116

[B42] Zhao G. Lu H. Chang Z. Zhao Y. Zhu T. Chang L. (2021). Single-cell RNA sequencing reveals the cellular heterogeneity of aneurysmal infrarenal abdominal aorta. Cardiovasc Res. 117, 1402–1416. 10.1093/cvr/cvaa214 32678909 PMC8064434

